# Advances and Applications of Block Copolymers II

**DOI:** 10.3390/polym18030335

**Published:** 2026-01-27

**Authors:** Nikolaos Politakos, Apostolos Avgeropoulos

**Affiliations:** 1 Institute of Chemical Biology, National Hellenic Research Foundation, 11635 Athens, Greece; 2 Department of Materials Science Engineering, University of Ioannina, 45110 Ioannina, Greece

Polymers are an essential class of materials; they are constantly evolving, and new monomers/polymers are being synthesized and/or produced every day. Polymeric materials can be used in both every-day and cutting-edge applications, including nanotechnology, energy, biotechnology, environment, aerospace, electronics, and agriculture industries. Currently, enormous challenges are being raised where polymers may offer solutions. Globally, there is a focus on circular economy and sustainability, high-performance and functional materials, and advanced manufacturing.

Block copolymers (BCPs) are a major part of polymeric materials overall. This class of materials is evolving through the development of new monomers and polymerization techniques, leading to biodegradable BCPs for novel applications. Self-assembly of BCPs can lead to improved applications in nanolithography and nanomanufacturing, as well as in various hierarchical architectures. In energy storage and conversion, particularly in photovoltaics, solid polymer electrolytes and BCPs are also essential. Another growing area of research into BCPs is innovative nanomedicine and targeted delivery of bioactive compounds, in which stimulus-responsive systems based on BCPs are being synthesized to deliver drugs, RNA, or proteins with high accuracy. The use of BCPs as vaccine platforms or in bioimaging is also relatively novel and currently under development. Finally, BCPs are also important for sustainability applications, especially as bio-based materials.

However, BCPs face new challenges every day, and scientists should focus their research on current and future trends in applications. Active areas of research include semiconductors, high-energy solid-state batteries, chemical recycling, personalized medical treatments, conversion of toxic materials, reutilization, and membranes.

This Special Issue is a collection of scientific contributions from research groups investigating diverse copolymers, highlighting recent advances in polymers, functionalities, and applications. The publication of thirteen (13) manuscripts in this Special Issue underscores the significance of block copolymers (BCPs) and demonstrates the sustained interest of the scientific community in the development and application of these materials.

A study on the compatibility, crystal structure, and mechanical properties of new-generation block copolymer PP-b-PE (polypropylene-b-polyethylene) and PPR (random polypropylene) blends has revealed that PPR crystallizes first, during the cooling process, inducing early crystallization of PP-b-PE, which increases the crystallization temperature of PPR/PP-b-PE blends [1].

Another study has explored the self-assembly of cylinder-forming BCPs under the guidance of pillar topographic patterns, utilizing mesoscale density functional theory simulations. This investigation also focuses on directed self-assembly structures of cylinder-forming BCPs with varying parameters, including pillar pitch and diameter. The insights from this study provide a theoretical framework for designing directed self-assembly systems capable of multiplying the density of contact holes, a crucial component of lithographic processes used in electronic devices [2].

Moreover, a series of novel renewable copolymers based on poly(ethylene succinate) (PESu) and poly(isosorbide succinate) (PISSu), with a varying isosorbide (Is)/PESu molar ratio, were synthesized in situ and studied. The designed copolymers were characterized in depth with respect to their structural and thermodynamic properties. Dielectric spectroscopy, along with proper analysis, enabled molecular dynamics mapping of both local and segmental types, which is presented for these materials for the first time. Based on the overall findings, the systems were homogeneous, with the two polymer segments thoroughly distributed. Upon analyzing the results, evaluating them by using widely adopted routes and models, and comparing those from different techniques, an in-depth structure–property relationship was found [3].

Other experimental work focused on interpenetrating hydrogel networks (IHNs) composed of p(HEMA) or p(MAA) and different pluronic block copolymers that were designed for potential use as medical device biomaterials or as platforms for controlled drug delivery. In this study, the physicochemical properties of IHNs provided a greater understanding of the physical state of the two polymer components as well as the interactions between them, and how these interactions affect retention within the network when immersed in an aqueous fluid. This approach provided invaluable knowledge of the practical uses of these specific IHNs as medical device biomaterials/coatings [4].

The high importance of BCPs in structural biology is evident in the production of functionally active membrane proteins (MPs) in an appropriate membrane environment. Polymer–lipid particles based on styrene and maleic acid (SMA) represent a promising type of membrane mimic, as they can extract properly folded MPs directly from their native lipid environment. The introduction of a novel SMA derivative with a negatively charged taurine moiety demonstrates that both polymers can form nanodiscs with a patch of lipid bilayer that can undergo phase transitions at temperatures close to those of the lipid bilayer membranes [5].

In the field of thermal insulation, a study of melamine–aniline–formaldehyde copolymer organoclay nanocomposite foams is presented, analyzing their structural and textural properties and investigating their thermal insulation and strength. Better thermal insulation behavior indicates the effect of a more uniformly packed microsphere arrangement and a more elastic network structure, including aniline–formaldehyde bridges, on the formation of void structures. The thermal insulation values decreased gradually with increasing organoclay content in resin–organoclay nanocomposites, while the mechanical stability indicated an increase in elasticity caused by the incorporation of aniline–formaldehyde bridges into the polymer network [6].

There is global demand for stimuli-responsive polymeric nanostructures for a wide range of applications. Self-assembling amphiphilic copolymers with stimuli-responsive characteristics—created by introducing a hydrophilic tuneable monomer, (2-dimethylamino)ethyl methacrylate (DMAEMA), together with a hydrophilic one, lauryl methacrylate (LMA)—within linear and branched copolymer topologies were investigated. The structural changes in the obtained self-organized supramolecular structures were thoroughly analyzed using aqueous media with varying pH and salinity values. These findings contribute to general knowledge of the synthesis of linear and hyperbranched amphiphilic polyelectrolyte-type copolymers and their self-assembly behavior, leading to the formation of nanostructures in solution. The pH- and salt-responsive properties of the PDMAEMA component, together with variations in copolymer composition, allow for fine-tuning of the formed nanoparticles’ structures [7].

Additionally, responsive material and environmental applications could be possible for the CO_2_-responsive polymer poly(N-[3-(dimethylamino)propyl]-acrylamide)-b-poly(methyl methacrylate) (PDMAPAm-*b*-PMMA), which is expected to be implemented in direct air capture (DAC) technology. In this work, a kinetic model, a crucial tool for optimizing polymer synthesis protocols and facilitating scaled-up production of the above-mentioned copolymer, is presented. In the end, the optimal parameters were determined for synthesizing a CO_2_-responsive diblock copolymer of PDMAPAm-b-PMMA with the desired molar mass and PDMAPAm block-to-PMMA block molar mass ratio [8].

The mechanical properties of double hydrophilic block copolymers based on poly[oligo(ethylene glycol) methacrylate] (POEGMA) and poly(vinyl benzyl trimethylammonium chloride) (PVBTMAC) blocks were studied using small-amplitude oscillatory shear rheology. The authors showed that changing the block arrangement along the backbone from statistical to sequential results in a distinct change in the viscoelastic response, indicating the presence/absence of bulk-like regions. The tunable viscosity and shear-thinning behavior achieved by altering the copolymer composition and block arrangement along the backbone render these materials promising candidates for drug delivery applications. Overall, this study provides information about the viscoelastic properties of densely grafted macromolecular architectures and shows how the mechanical and dynamic properties can be tailored for different drug delivery applications by simply altering the ratio between the two homopolymers [9].

Further work on the combination of Brownian dynamics simulations and self-consistent field theory has demonstrated that stable assembled structures, such as vesicles, toroidal micelles, bowl-like micelles, sheet-like micelles, non-spherical vesicles, and cylindrical micelles, are dependent on the molecular parameters of amphiphilic comb-like copolymers. In this study, it was found that vesicle formation involves two intermediate states—sheet- and bowl-like micelles—and that the difference in their free energies is minimal, indicating coexistence. This study describes how to obtain different morphologies by adjusting the molecular parameters of amphiphilic comb-like copolymers, further confirming their potential for stable drug encapsulation and enhanced targeted drug delivery [10].

In the field of self-assembly behavior, a series of amphiphilic diblock copolymers, each consisting of a hydrophilic poly(N-vinyl pyrrolidone) (PNVP) block and a hydrophobic block derived from n-alkyl vinyl esters, namely, poly(vinyl butyrate) (PVBu), poly(vinyl decanoate) (PVDc), and poly(vinyl stearate) (PVSt), in aqueous solutions was investigated. Using dynamic and static light scattering techniques, the micellization behavior was studied. The effect of the hydrophobic block’s nature in the copolymer composition, as well as the copolymer molecular weight on the self-assembly properties were thoroughly examined. The study also investigated the encapsulation of curcumin and indomethacin within the dry cores of the micellar structures. Combined with the observed stability of the micellar structures, these findings suggest that block copolymers have significant potential as drug carriers [11].

Another study on the self-assembly of a high-molecular-weight poly(styrene-*b*-isoprene) block copolymer was analyzed after chemical modification. The polymer was converted into poly(styrene-*b*-(ethylene-alt-propylene)) by hydrogenation and into poly(styrene-*b*-sulfonated isoprene) by mild sulfonation of the PI block. The obtained morphologies were examined by atomic force microscopy to analyze the effects of sample preparation parameters, including solvent, casting technique, and annealing temperature, which highlighted that significant morphological and topographical changes were observed across different parameters used. Each modification introduces new variables that can affect the final structure and properties of different types of copolymers [12].

Finally, a review of the different routes and methods for preparing hybrid materials based on nanostructured block copolymers (BCPs) and magnetic nanoparticles (MNPs) is included in this Special Issue. This work synthesizes studies on the synthesis and characterization of BCP/MNP hybrid systems using various strategies or methods, highlighting the main findings and potential applications of the resulting materials [13].

Throughout this Special Issue, it is clear that block copolymers play a key role in advancing polymer science. The progress of block copolymers, both in synthesis and in self-assembly, as well as diverse applications, is thoroughly demonstrated in this collection. As Guest Editors, we are confident that these studies will make a substantial contribution to the field of block copolymers.
